# Friction phenomena and their impact on the shear behaviour of granular material

**DOI:** 10.1007/s40571-016-0119-2

**Published:** 2016-06-13

**Authors:** Bettina Suhr, Klaus Six

**Affiliations:** Virtual Vehicle Research Center, Inffeldgasse 21/A, 8010 Graz, Austria

**Keywords:** Friction, Tribology, Direct shear test, Granular materials, DEM

## Abstract

In the discrete element simulation of granular materials, the modelling of contacts is crucial for the prediction of the macroscopic material behaviour. From the tribological point of view, friction at contacts needs to be modelled carefully, as it depends on several factors, e.g. contact normal load or temperature to name only two. In discrete element method (DEM) simulations the usage of Coulomb’s law of friction is state of the art in modelling particle–particle contacts. Usually in Coulomb’s law, for all contacts only one constant coefficient of friction is used, which needs to reflect all tribological effects. Thus, whenever one of the influence factors of friction varies over a wide range, it can be expected that the usage of only one constant coefficient of friction in Coulomb’s law is an oversimplification of reality. For certain materials, e.g. steel, it is known that a dependency of the coefficient of friction on the contact normal load exists. A more tribological tangential contact law is implemented in DEM, where the interparticle friction coefficient depends on the averaged normal stress in the contact. Simulations of direct shear tests are conducted, using steel spheres of different size distributions. The strong influence of interparticle friction on the bulk friction is shown via a variation of the constant interparticle friction coefficient. Simulations with constant and stress-dependent interparticle friction are compared. For the stress-dependent interparticle friction, a normal stress dependency of the bulk friction is seen. In the literature, measurements of different granular materials and small normal loads also show a stress dependency of the bulk friction coefficient. With increasing applied normal stress, the bulk friction coefficient reduces both in the experiments and in the simulations.

## Introduction

Solid-like granular materials generally comprise a high number of particle–particle and particle–environment contacts with high variations in normal loading. The frictional behaviour of these contacts has a high influence on the macroscopic behaviour of the material. In the sense of a tribological system, friction is influenced by several parameters like contact normal load, relative motion, surface roughness, contact temperature, and contact conditions (dry, wet, lubricated contact conditions, etc.).

The discrete (distinct) element method (DEM) was introduced by Cundall and Strack, see [4], and has become a widely used tool for modelling the mechanical behaviour of solid-like granular materials. While there are several topics of active research focusing on the improvement of the prediction quality of DEM simulations, it is state of the art to consider the frictional behaviour of contacts by application of Coulomb’s law with one constant interparticle friction coefficient. At a contact, the resulting contact force is decomposed in normal and tangential direction, $F_\mathrm{n}$ and $F_\mathrm{t}$, and contact laws for their calculation are chosen. In tangential direction, the force, which can be transferred, is bounded. For cohesionless materials, Coulomb’s law can be written as follows:

1 $$\begin{aligned} F_\mathrm{t}=\min \left( \mu F_\mathrm{n}, \tilde{F_\mathrm{t}}\right) , \end{aligned}$$

where $\mu$ is the constant interparticle friction coefficient and $\tilde{F_\mathrm{t}}$ is the pre-sliding shear force calculated using the contact constitutive model. Coulomb’s law can also be stated using the internal friction angle, $\phi$, which is connected to the interparticle friction coefficient by $\mu =\tan (\phi )$.

Frequently used tests for the investigation of the bulk shear behaviour of granular materials are the triaxial test and the direct shear (or shear box) test. Usually, the Mohr–Coulomb failure criterion is used, which reads as

2 $$\begin{aligned} \tau _\mathrm{f}=\tan (\varPhi ) \sigma _\mathrm{n} + c, \end{aligned}$$

where $\tau _\mathrm{f}$ is the final shear stress, $\varPhi$ is the bulk friction angle and *c* is a material parameter representing cohesion of the granular material, i.e. $c=0$ for cohesionless materials. The bulk friction angle of a granular material is an important characteristic for its shear behaviour. Alternatively, the peak friction angle can be determined, where the maximal shear stress instead of the final one is used in Eq. (2).

In the literature there exist several works, which state a strong influence of the interparticle friction on the bulk friction angle, see, e.g. [3, 7] or [11], who simulated direct shear tests and compared the results to experiments.

Also, it is frequently found in the literature that the bulk/peak friction angle of a granular material is constant, i.e. independent of the normal stress. It is assumed by the authors of this work that two aspects contribute to this conclusion. On the one hand, often very small equi-sized spherical particles are considered. Here, the dependency of the bulk friction coefficient on the normal stress is usually negligible. On the other hand, the way of analysing the results can sometimes be misleading. In the frequently found plot, shear stress over normal stress, it is very hard to see deviations from the linear trend. To investigate a stress dependency of the bulk friction coefficient other representations can be more helpful, e.g. bulk friction over normal stress or bulk friction over porosity.

Two examples for the shear behaviour of equi-sized spheres were found by Cui and O’Sullivan [3], and Härtl and Ooi [7]. Direct shear tests with different normal stresses were conducted on equi-sized steel balls and glass beads, respectively. In the regime of applied normal stresses, in both works a linear relation between the measured shear stress and normal stress was found. Thus, the application of the Mohr–Coulomb criterion was justified and the bulk friction angle was constant.

On the contrary, different results are obtained by Härtl and Ooi [7], when the shear test is performed on paired glass beads (two beads glued together) instead of single glass beads. Here, the applied normal stress ranges from 3 to 24 kPa, and a dependency of the bulk friction angle on these stresses can be seen. In [8], Härtl and Ooi compare the same experimental results to DEM simulations. The stress dependency of the bulk friction angle, found in the experimental results, could not be reproduced in the DEM simulations, which used a constant interparticle friction coefficient for all load cases.

Similar experimental results regarding a stress dependency of the bulk friction angle are found from Indraratna et al. in [9], where railway ballast is investigated in direct shear tests. The normal stress is varied between 15 and 75 kPa, and a non-linear dependency between shear stress and normal stress is shown. Here, also several works on rock-fill materials are cited, which state a non-linear relationship, which is significant at low normal stresses and gradually reduces as the normal stress increases.

This description matches well with the results of Tuzun and Walton [18]. A stress-dependent coefficient of friction between smooth silo walls and particles was found for small normal stresses. It seems that depending on the considered material and particle shape, a non-linear relation between shear stress and normal stress can be observed for low normal stresses.

**Fig. 1:**
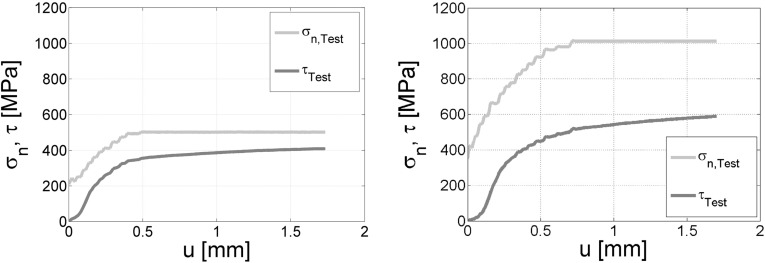
High Pressure Torsion (HPT) tests, where two steel discs are rotated against each other. *Left* normal stress $\sigma _\mathrm{n}$ = 500 MPa. *Right* normal stress $\sigma _\mathrm{n}$ = 1000 MPa. Measurement of normal stress ($\sigma _\mathrm{n}$) and shear stress ($\tau$) over displacement *u*; increasing normal stress reduces ratio $\tau$/$\sigma _\mathrm{n}$

Motivated by the above experimental findings on granular media and results obtained on the wheel–rail contact for steel, the authors will use a non-constant coefficient of friction in DEM simulations of direct shear tests on steel spheres (particle–particle contact). This paper is organised as follows. In Sect. 2 an experimental motivation for the stress dependency of the friction coefficient will be given as well as the modified DEM contact model with stress dependency of the friction coefficient. The following Section contains details of the simulated direct shear tests. Samples of three different size distributions will be considered and differences between simulations with constant and stress-dependent interparticle friction coefficient will be presented. Finally, conclusions will be drawn in Sect. 4.

## Stress-dependent friction coefficient

### Experimental motivation

Coulombs law is usually used with one constant friction coefficient. It is known in tribology that this is not enough to model frictional contacts under some conditions. In many experiments, the observed frictional behaviour is severely influenced by the applied normal stress, sliding velocity, temperature, surface conditions as well as other factors. Further reading for these general phenomena can be found, e.g. in [10]. For several materials the normal stress dependency of the friction coefficient is reported in the literature, e.g. for aluminium or polymers, see, e.g. [2, 13], and for E-glass fibre-reinforced epoxy composites, see [5]. The authors of this work were motivated to introduce a stress-dependent friction coefficient by works on wheel–rail contacts (steel–steel). In [14], experimental results could not be reproduced using a constant coefficient of friction. In Fig. 1 results of High Pressure Torsion tests (HPT) are shown. In a HPT test two steel discs are rotated against each other, while the normal stress, $\sigma _\mathrm{n}$, and the shear stress, $\tau$, are measured. In this case, the ratio between $\tau$ and $\sigma _\mathrm{n}$ is the coefficient of friction. From the left to the right plot, the maximum normal stress $\sigma _\mathrm{n}$ is doubled. If the coefficient of friction was constant, then $\frac{\tau }{\sigma _\mathrm{n}}$ would be constant and thus $\tau$ would be doubled. In right plot of Fig. 1 it can be seen that the $\tau$ is clearly lower, and therefore, a significant dependency of the coefficient of friction on the normal load can be concluded from the experiments.

**Fig. 2:**
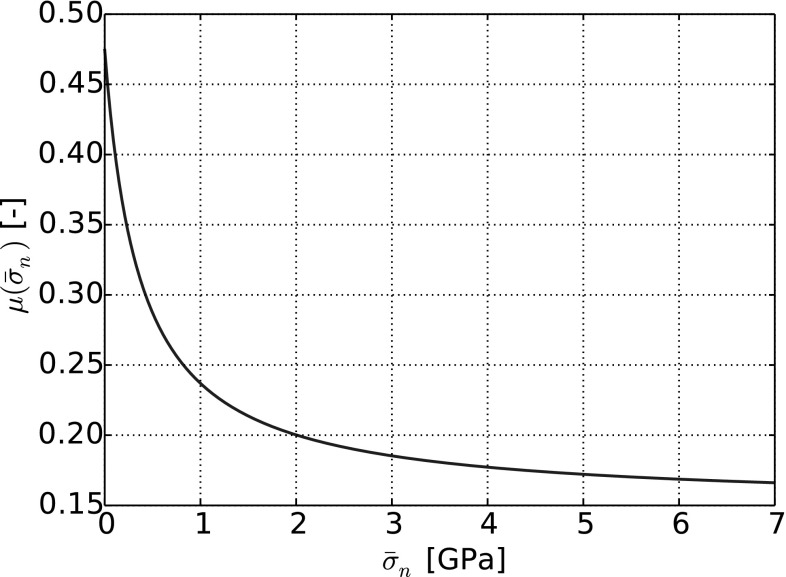
Graph of the stress-dependent coefficient of friction as defined in Eq. (3)

Moreover, in the European Standard [6], a normal load dependency of the friction coefficient in wheel–rail contact is given based on measurements on a wheel–rail test rig. Matching to the described findings are the results obtained in [12] from Popov et al. With the method of Movable Cellular Automata (MCA), the wheel–rail contact is modelled, (steel–steel) and from simulation results a normal stress-dependent coefficient of friction is derived:

3 $$\begin{aligned} \mu (\sigma _\mathrm{n})=0.15 + \frac{0.3243}{1+0.00212 \frac{\sigma _\mathrm{n} E}{\sigma _0^2}}, \end{aligned}$$

where $\sigma _\mathrm{n}$ is the applied normal stress, *E* is the Young modulus, $E=206$ GPa, and $\sigma _0$ is the ultimate strength and was varied between 92 and 552 MPa. The graph of the above function is plotted in Fig. 2 for $\sigma _0= 400$ MPa. For low normal stress the value of the friction coefficient decays non-linearly and approaches an asymptotic value for high normal stresses. Thus, for high normal stresses the proposed model coincides with the constant friction coefficient, often used in DEM simulations.

The stress-dependent coefficient of friction, (3) from Popov et al. [12], will be used in a DEM contact model, which is described in detail in the next subsection. In [16], Suhr and Six used the same contact model (with different parameters) to simulate direct shear tests on single and paired glass beads, as conducted by Härtl and Ooi, see [8]. Here, ‘single glass spheres’ refers to equi-sized spheres, while ‘paired spheres’ refers to two equi-sized spheres, which are glued together. For glass–glass contacts, no data were available for the parameters of the stress-dependent coefficient of friction in Eq. (3). A parameter study was conducted, and simulation results for the bulk behaviour were in good accordance with the experimental results as described in [8]. For single spheres the bulk friction angle was nearly constant, while for paired spheres a clear normal stress dependency could be seen in simulations as well as in experiments. In the present work, the parameters of the stress-dependent coefficient of friction for steel–steel contacts are taken from Popov et al. [12]. It will be future work to obtain experimental data for the direct shear tests for a comparison with the simulated bulk behaviour.

### DEM contact model with stress-dependent friction coefficient

In the following DEM simulations, the simplified Hertz Mindlin contact model without microslip and with damping as described by Tsuji et al. [17], and Antypov et al. [1], will be used. In normal direction of the contact, the Hertz model is as follows:

4 $$\begin{aligned} F_\mathrm{n}=\frac{4}{3} \hat{E} \sqrt{\hat{R}} \sqrt{u_\mathrm{n}^3}, \end{aligned}$$

where $\hat{E}$ is the equivalent Young modulus of the contact, $\hat{R}$ is the equivalent contact radius and $u_\mathrm{n}$ is the overlap in normal direction. In the Hertzian contact model the area of contact is circular (sphere–sphere contact). The spatial distribution of the stress over the contact area is also known, but will not be used. Instead, for each single contact a (spatially) averaged stress, $\bar{\sigma }_\mathrm{n}$, can be calculated by dividing the contact force by the contact area:

5 $$\begin{aligned} \bar{\sigma }_\mathrm{n}:=\frac{F_\mathrm{n}}{a^2 \pi }= \frac{F_\mathrm{n}}{\pi } \left( \frac{4\hat{E}}{3F_\mathrm{n} \hat{R}}\right) ^{\frac{2}{3}}, \end{aligned}$$

where $a = \left( \frac{3F_\mathrm{n} \hat{R}}{4\hat{E}}\right) ^{\frac{1}{3}}$ is the radius of the contact patch. In the tangential direction of the contact, the Mindlin model without microslip is applied. In time step *k* the trial or pre-sliding shear force is denoted by $F_\mathrm{t,t}^k$ and is calculated incrementally using the last time step’s value, $F_\mathrm{t}^{k-1}$:

6 $$\begin{aligned} F_\mathrm{t,t}^k = F_\mathrm{t}^{k-1} + \varDelta F_\mathrm{t,t} \quad \varDelta F_\mathrm{t,t} = 8\, a\, \hat{G} \, \varDelta u_\mathrm{s}, \end{aligned}$$

where $\hat{G}$ is the equivalent shear modulus and $\varDelta u_\mathrm{s}$ the increment of the shear displacement. For brevity the index of the time step will be dropped from now on. Using the constant coefficient of friction, the shear force is given by

7 $$\begin{aligned} F_\mathrm{t}=\left\{ \begin{array}{ll} F_\mathrm{t,t}&{} \quad \text{ if }\; F_\mathrm{t,t}\le \mu F_\mathrm{n}\\ \mu F_\mathrm{n}&{} \quad \text{ otherwise } \end{array} \right. . \end{aligned}$$

For the use of the stress-dependent friction coefficient, we now change Eq. (7) to

8 $$\begin{aligned} F_\mathrm{t}=\left\{ \begin{array}{ll} F_\mathrm{t,t}&{} \quad \text{ if }\; F_\mathrm{t,t}\le \mu (\bar{\sigma }_\mathrm{n}) F_\mathrm{n}\\ \mu (\bar{\sigma }_\mathrm{n}) F_\mathrm{n}&{} \quad \text{ otherwise } \end{array} \right. , \end{aligned}$$

where $\bar{\sigma }_\mathrm{n}$ is given by Eq. (5) and $\mu (\bar{\sigma }_\mathrm{n})$ by Eq. (3) from Popov et al. [12]. Here, it is important to note that the averaged stress, $\bar{\sigma }_\mathrm{n}$, is the spatial average of the stress at one single contact and not the average over several contacts. In the proposed model each contact is treated individually. After the computation of normal and tangential force for each contact, the damping is applied as described by Tsuji et al. [17], and Antypov and Elliott [1].

In the previous explanation, the stress-dependent interparticle friction coefficient is combined with Hertz–Mindlin contact law. However, its use is not restricted to this contact law or spherical particles, but it can be combined with other particle shapes and any other contact law, which uses Coulomb’s law in tangential direction. The easiest way is to define an area of contact for the contacting bodies, e.g. sphere–sphere or polyhedron–polyhedron. Then, the averaged stress can be computed as described above. For complex, irregular particles shapes no analytical expression for the area of contact exists. An approximation could be made by relating the overlap (distance or volume), calculated in the contact detection phase, with a particle shape-dependent multiplier. For spheres and the Hertz–Mindlin contact law, the contact area is given by $\pi \, \hat{R} \, u_\mathrm{n}$. By choosing an averaged equivalent contact radius, $\hat{R}$, for the considered granular material, an approximation of the contact area for contacting spherical bodies can be made. For other particles shapes, different choices will have to be made. A second possibility, for using the proposed approach with non-spherical particles, would be to make the friction coefficient dependent on the contact force instead of the contact stress. Of course, a calibration and validation of the chosen approach is necessary, also.

## DEM simulation of direct shear tests

The influence of interparticle friction on the macroscopic behaviour of a granular material will be investigated via simulation of direct shear tests. Results of a variation of the constant friction coefficient will be compared to those obtained with the above introduced stress-dependent friction coefficient.

All simulations are conducted with the DEM software Yade, [15]. In this software the soft contact approach is used together with explicit discretisation in time. As already mentioned, the main focus of this work is the modification of the tangential contact law, regarding the friction coefficient. The basis for this work is the simplified Hertz–Mindlin model without microslip as given in Eqs. (4, 7). If the modified contact law, Eqs. (4, 8), is used, it will be stated explicitly.

**Fig. 3:**
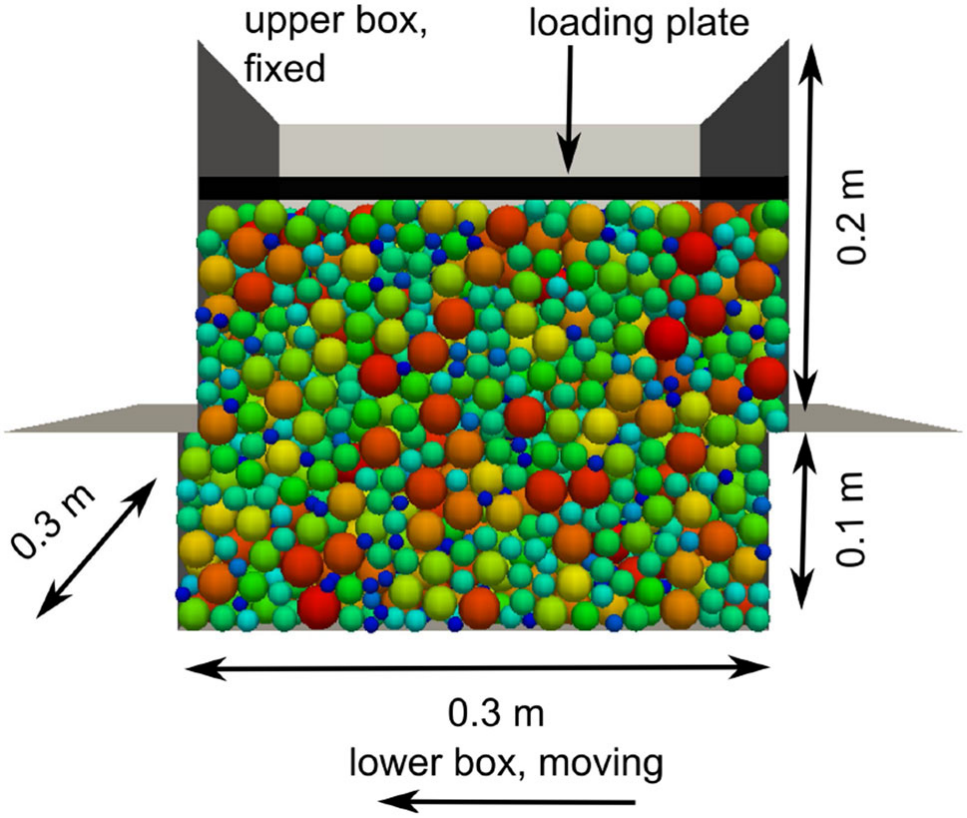
Setup of shear box test

**Fig. 4:**
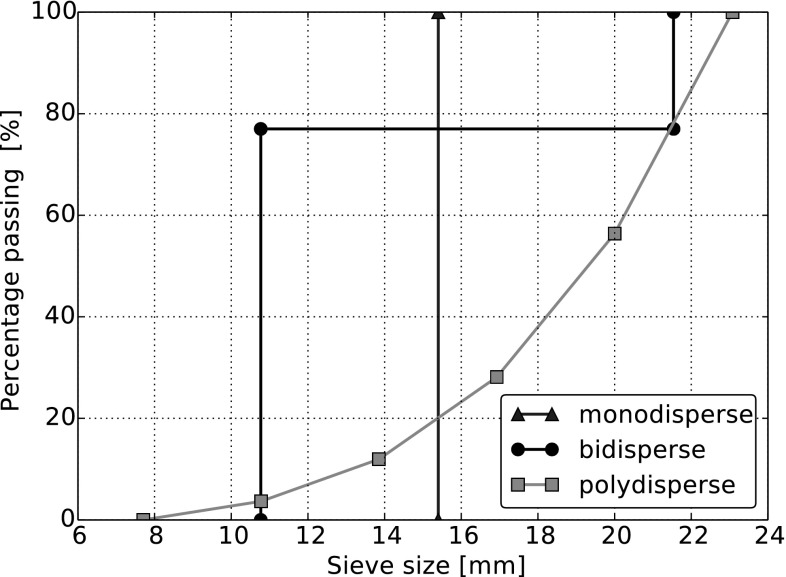
Size distribution of spheres used for testing

Samples consisting of three different size distributions of steel spheres are used for direct shear tests. The setup of such a direct shear test is shown in Fig. 3. The lower box has the dimensions 0.3 m $\times$ 0.3 m $\times$ 0.1 m and the upper box 0.3 m $\times$ 0.3 m $\times$ 0.2 m. In Fig. 4 the size distribution of the used samples are shown: monodisperse samples (equi-sized spheres), bidisperse samples (two sizes of spheres) and polydisperse samples (given size gradation) are considered. It will be investigated, if the effect of the stress-dependent interparticle friction is different for samples with different size distributions. The material of the spheres and the walls of the shear box is assumed to be equal. The material parameters of steel used in the DEM simulation are summarised in Table 1.

**Table 1:** Parameters used in DEM simulations

Parameter	Density (kg/m$^3$)	Young modulus (GPa)	Poisson’s ratio
Value	7833.34	200	0.28

The sample generation differs for the three different size distributions. For monodisperse samples, 6000 spheres of diameter 15.4 mm are randomly placed above the shear box and are allowed to settle inside the box. To investigate the influence of the initial configuration, five different samples are generated, which vary in height between 0.217 m and 0.221 m. The bidisperse samples are built in three steps. At first, 8000 spheres of diameter 21.5 mm are allowed to settle inside the box. Then, the diameter of three quarters of the spheres is reduced to 10.8 mm and the sample is again allowed to settle. To achieve a uniform height of all samples, all spheres above 0.22 m are erased. The five produced samples consisted of 7287–7418 spheres with 77 % small spheres and 23 % large spheres. A two-stage procedure is used to generate polydisperse samples. First, 6000 spheres, whose diameter vary between 7.7 and 23 mm, are allowed to settle inside the box. In the second step, the sample is again cut at 0.22 m height. Five different samples were generated, which vary in particle number between 5031 and 5083. The samples had the shown gradation with minimal deviations. To achieve dense packings, the friction coefficient is set to 0 and gravity is increased by factor 5 in all cases until the samples have finally settled. In the next step, a steel plate is inserted above the spheres, the friction coefficient is set to its defined value, which is 0.2 for a start, and gravity is reduced to 9.81 m/s$^2$. Now, the normal load is applied on the spheres using a servo control mechanism (P-control). After the specified normal load is reached and the spheres are at rest, the shearing phase starts by imposing a velocity on the lower shear box. Variations of the shear velocity showed that shearing with 10 mm/s yielded results which can be considered quasi-static, i.e. a lower shearing rate yielded the same result. During the simulations, the applied normal load could be controlled with an error below 2 %.

**Fig. 5:**
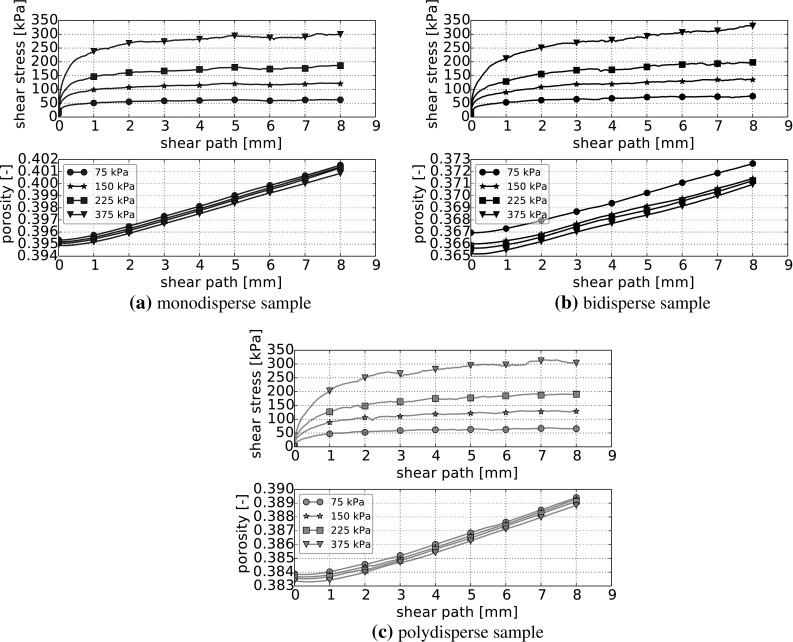
Simulation results for direct shear tests of samples with constant interparticle friction $\mu =0.2$. Shear stress and porosity over shear displacement for the four levels of applied normal stress

The direct shear test will be simulated with four different levels of applied normal stress, $\sigma _\mathrm{n} = 75$, 150, 225 and 375 kPa. At first, the interparticle friction coefficient is constant, $\mu =0.2$, and the simplified Hertz–Mindlin model (4, 7) is used. In Fig. 5, the shear stress over the shear path is shown in the upper plots for the three different particle size distributions. For the calculation of the shear stress, all contact forces belonging to the lower box and the bottom are summed; then only the component in shear direction is divided by the cross-sectional area of the shear box 0.09 m$^2$. For the three size distributions, the shear forces belonging to the different applied normal stresses are in a similar range. In the lower plots of Fig. 5, the porosity of the samples is plotted over the shear path. At the beginning of all tests, there is a short phase where the samples are compacted, while dilation occurs for the rest of the simulation. The densest packings are obtained for the bidisperse sample, followed by the polydisperse sample and the loosest is the monodisperse sample.

**Fig. 6:**
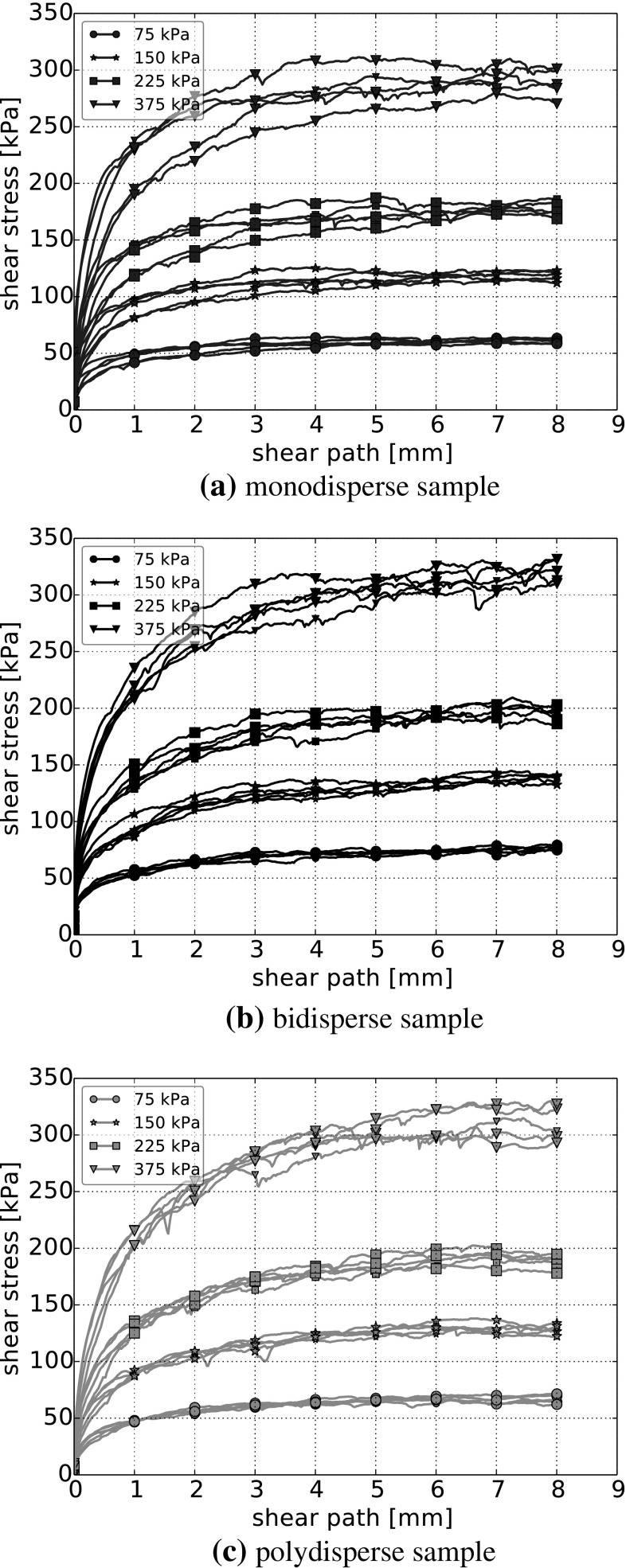
Influence of initial configuration: shear stress over shear path for five initial settings

To check the influence of the samples initial configuration on the simulation results, five different configurations were generated for each particle size distribution. The results can be seen in Fig. 6. Deviations towards the median of the final shear stress occur up to 5 % (monodisperse), 5 % (bidisperse) and 6 % (polydisperse). Similar results can be expected, if experiments in the lab were conducted. For the time being, influences off the different initial settings will be reported, where they are of interest. Also, differences in the initial porosity of the settings occur. For monodisperse samples the initial porosity lies between 0.394 and 0.396, for bidisperse samples between 0.364 and 0.368 and for samples with size gradation between 0.383 and 0.386.

Despite the considerable differences in the particle size distribution and the resulting differences in the sample’s porosity, the shear stress response of mono-, bi- and polydisperse samples is very similar for simulations with $\mu =0.2$. This is surprising as the bidisperse sample consists of 77 % of spheres with a considerably smaller radius than the spheres of the monodisperse sample. For monodisperse samples of smaller spheres the resulting shear stress is expected to be smaller as well. Thus, for the bidisperse sample the 23 % of spheres with a bigger radius play a key role for the bulk behaviour. A similar effect can be expected to be present in the polydisperse sample.

**Fig. 7:**
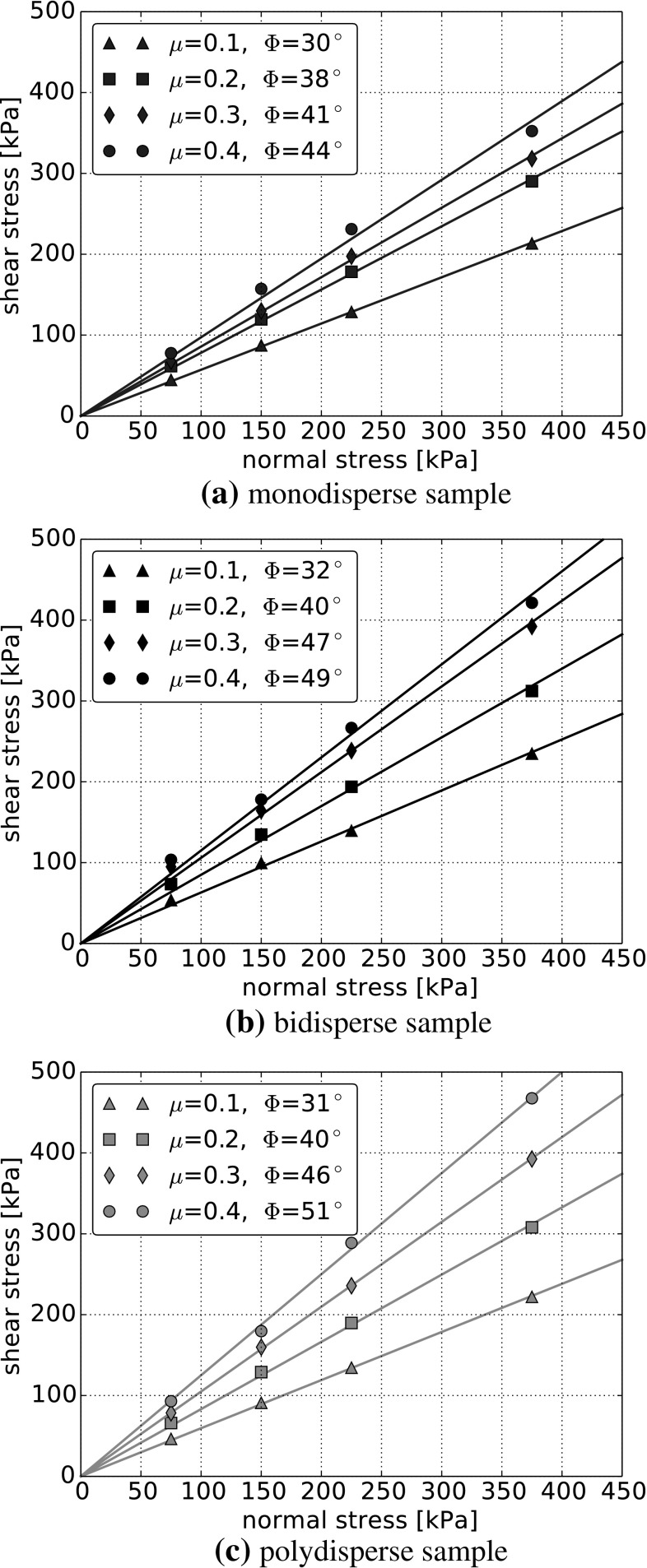
Influence of (constant) interparticle friction, $\mu$, on bulk friction. Final shear stress, $\tau _\mathrm{f}$, over applied normal stress, $\sigma _\mathrm{n}$

### Influence of constant interparticle friction coefficient

It is well known that interparticle friction is a key factor for the shear behaviour of granular materials. In the following, the interparticle friction coefficient will be varied between 0.1 and 0.4 to investigate its influence on the bulk friction angle for one considered initial setting for each particle size distribution. From the conducted simulations, the final shear stress is calculated as the median of the last hundred readings of the shear stress (over a shear path of 2 mm). Here, the median instead of the mean value is chosen due to its insensitivity with respect to outliers. Figure 7 shows the resulting final shear stresses, $\tau _\mathrm{f}$, over the applied normal stress, $\sigma _\mathrm{n}$, for the different values of $\mu$. The already mentioned Mohr–Coulomb criterion for cohesionless material is used, and the least squares fit for each value of interparticle friction is shown. The slope of these lines is the bulk friction coefficient and via the arc tangent the bulk friction angle, $\varPhi$, is calculated.

The Mohr–Coulomb criterion states a linear relation between normal stress and final shear stress. In the plots, no stress dependency of the bulk friction coefficient can be seen. For the monodisperse and polydisperse sample, the linear fit through the origin (cohesionless material) agrees reasonably well with the simulation data. The bidisperse sample shows some deviations for the smallest force level. A linear fit for material with cohesion probably would match the results better.

For low values of the interparticle friction coefficient, $\mu =0.1$ and $\mu =0.2$, the resulting bulk friction angles are similar for the mono-, bi- and polydisperse samples. For higher values of $\mu$ the bidisperse and polydisperse samples have still similar bulk friction angles, while the monodisperse sample has noticeably lower bulk friction angles.

### Usage of stress-dependent interparticle friction

**Fig. 8:**
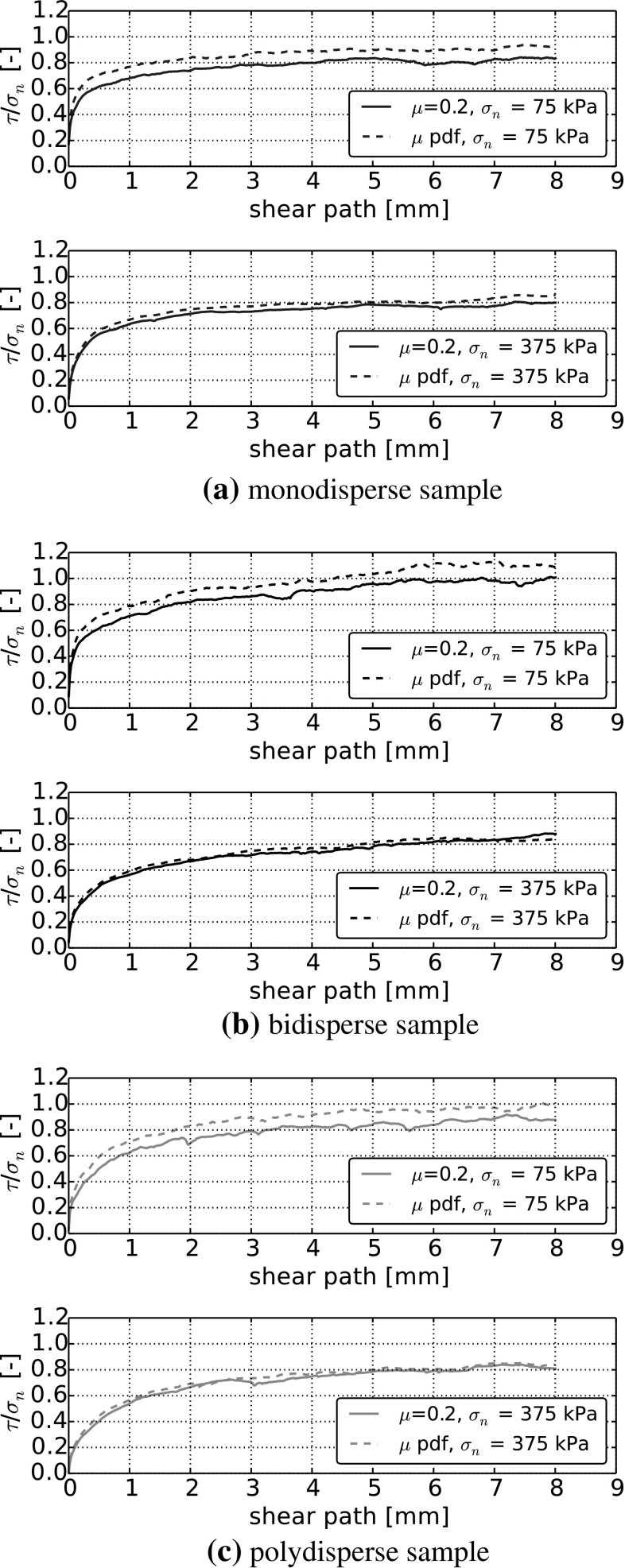
Comparison of constant interparticle friction and stress-dependent friction (pdf). Normalised shear stress over shear path. Comparison for $\sigma _\mathrm{n}=375$ kPa and $\sigma _\mathrm{n}=75$ kPa

As a next step, simulations with the stress-dependent interparticle friction coefficient, (3) from [12], together with the modified shear force law (8) are presented. The results will be compared to simulations using $\mu =0.2$, as values between 0.1 and 0.2 are frequently used for steel–steel contacts in literature. In the model of stress-dependent interparticle friction, the Young modulus is set to $E=200$ GPa. For the ultimate strength, $\sigma _0$, the interval of 92 to 552 MPa is specified in [12]. In this work, $\sigma _0=400$ MPa is used. In Fig. 8, the normalised shear stress, $\frac{\tau }{\sigma _\mathrm{n}}$, is plotted over the shear path for $\mu =0.2$ and the stress-dependent friction coefficient, denoted in the plot as $\mu$ pdf, for all three particle size distributions. Considering $\sigma _\mathrm{n}=375$ kPa in the lower subplots, then the simulation results for $\mu =0.2$ and stress-dependent $\mu$ agree well for all particle size distributions (calibration of the model via $\sigma _0$). In the upper subplots, where $\sigma _\mathrm{n}=75$ kPa, $\frac{\tau }{\sigma _\mathrm{n}}$ is about 10 % larger for stress-dependent $\mu$ than for $\mu =0.2$. For the stress-dependent $\mu$, the bulk friction coefficient decreases with increasing $\sigma _\mathrm{n}$ until it coincides at $\sigma _\mathrm{n}=375$ kPa with the value obtained with $\mu =0.2$, where it was calibrated, compare Fig. 8. This behaviour of the bulk friction coefficient qualitatively agrees with observations from direct shear experiments reported in the literature.

**Fig. 9:**
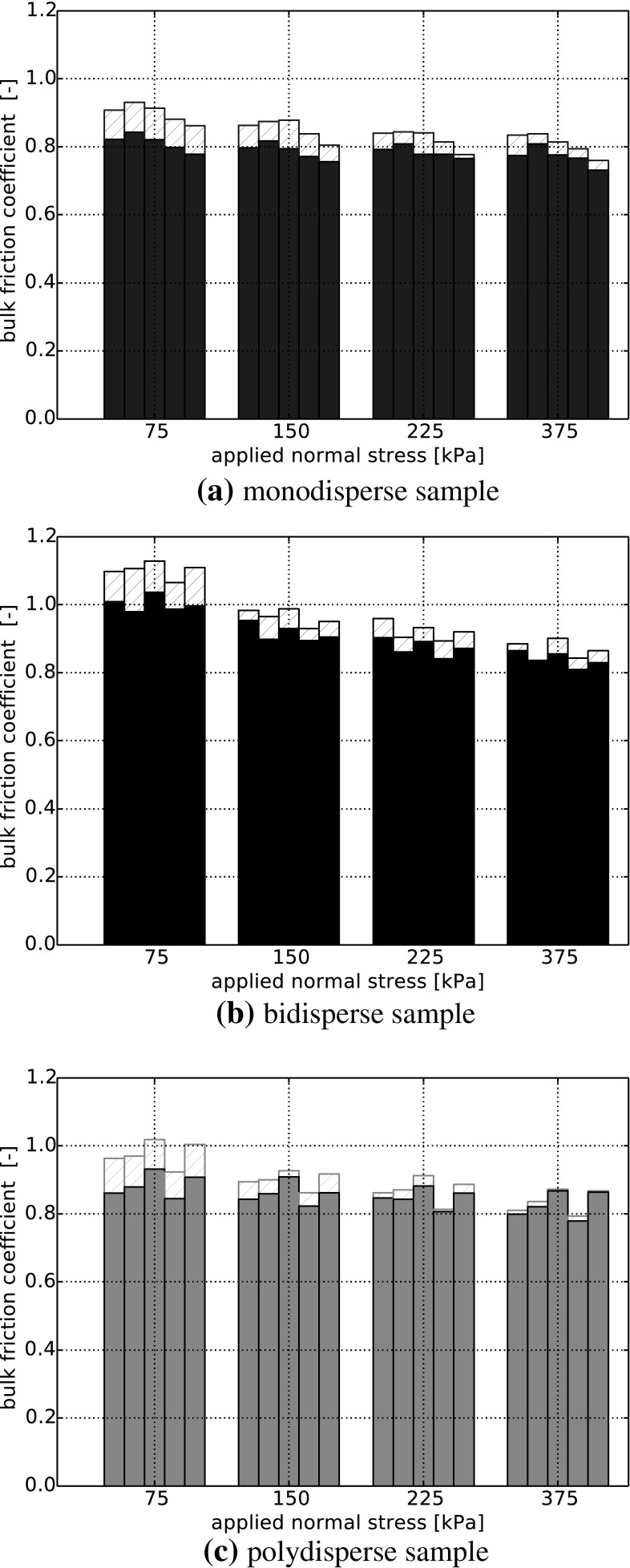
Final shear stress divided by normal stress over applied normal stress for five different initial settings. *Solid bars* $\mu =0.2$, *shaded bars* $\mu =$ pdf

The stress dependency in bulk friction, caused by the stress dependency in interparticle friction, is in a similar range as the scatter of results caused by the different initial settings, compare Fig. 6. A comparison of the simulations using $\mu =0.2$ and stress-dependent $\mu$ for all initial settings and applied normal loads is carried out. In Fig. 9 the bulk friction coefficient is calculated for each simulation individually. The solid bars show the bulk friction for the simulations with $\mu =0.2$ and shaded bars belong to stress-dependent $\mu$. For simulations with $\mu =0.2$, it can be seen from Fig. 9a, c that for monodisperse and polydisperse samples the bulk friction coefficient is nearly constant. For the highest and the lowest load level, the calculated values of the bulk friction coefficient vary only about 6 and 7 % for mono- and polydisperse samples, respectively. In contrast to this, there is a clear stress dependency seen in simulations of bidisperse samples with $\mu =0.2$, with about 20 % difference between bulk friction coefficients for the highest and the lowest load level. While this stress dependency is easy to see in Fig. 9b, it is hardly noticeable from widely used plots of shear force over shear stress, compare Fig. 7b.

The usage of stress-dependent interparticle friction in the simulations has a similar effect for the three different particle size distributions. For the lowest level of applied normal stress, the bulk friction is higher than values obtained with $\mu =0.2$. The bulk friction values decrease with increasing applied normal stress and are similar to the values obtained with $\mu =0.2$ for the highest level of applied normal stress. Between the highest and the lowest load level, the obtained values of the bulk friction coefficient vary about 11 % for monodisperse samples, about 27 % for bidisperse samples and about 17 % for polydisperse samples. From these results it can be seen that the extent of the stress dependency of the bulk friction coefficient differs for mono-, bi- and polydisperse samples, also scatter exists for the different initial settings. Nevertheless, the effect is present in all considered cases.

**Fig. 10:**
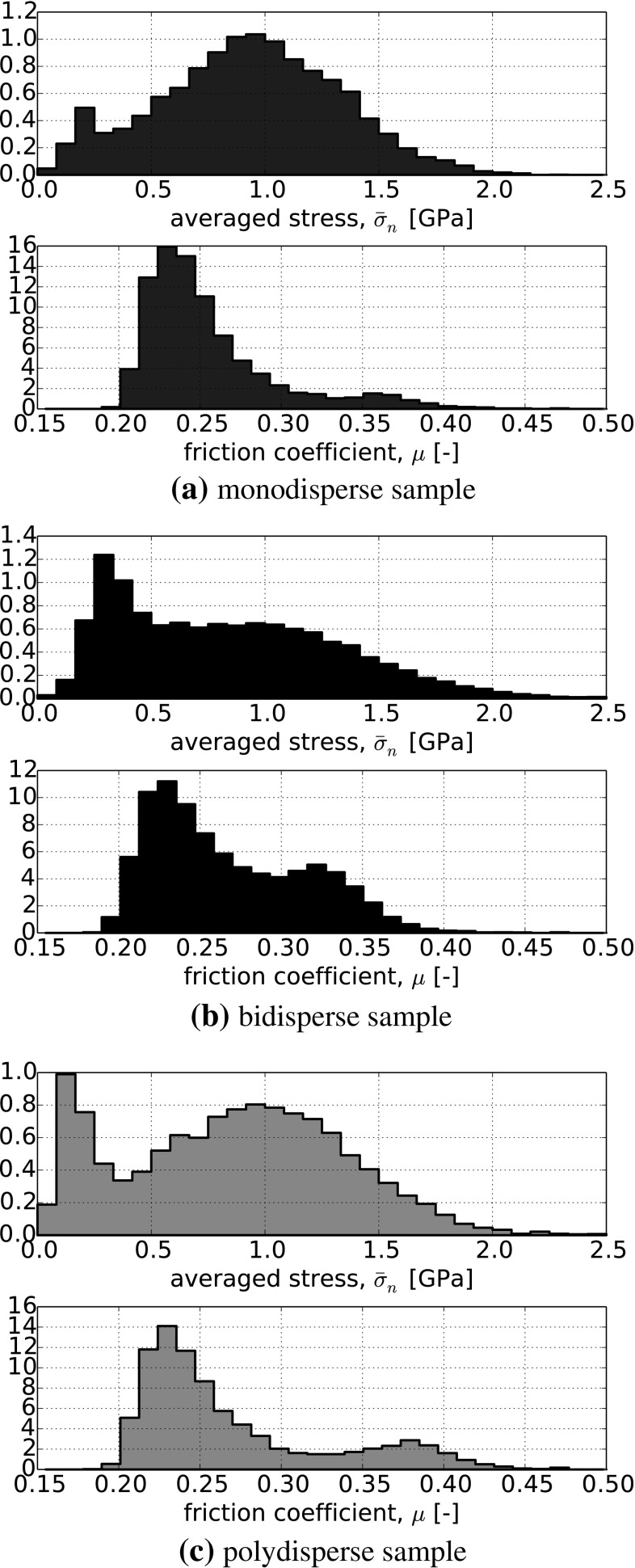
Simulations with stress-dependent friction coefficient at $\sigma _\mathrm{n}=75$ kPa: histogram of averaged stress, $\bar{\sigma }_\mathrm{n}$ (GPa) and stress-dependent $\mu$

**Fig. 11:**
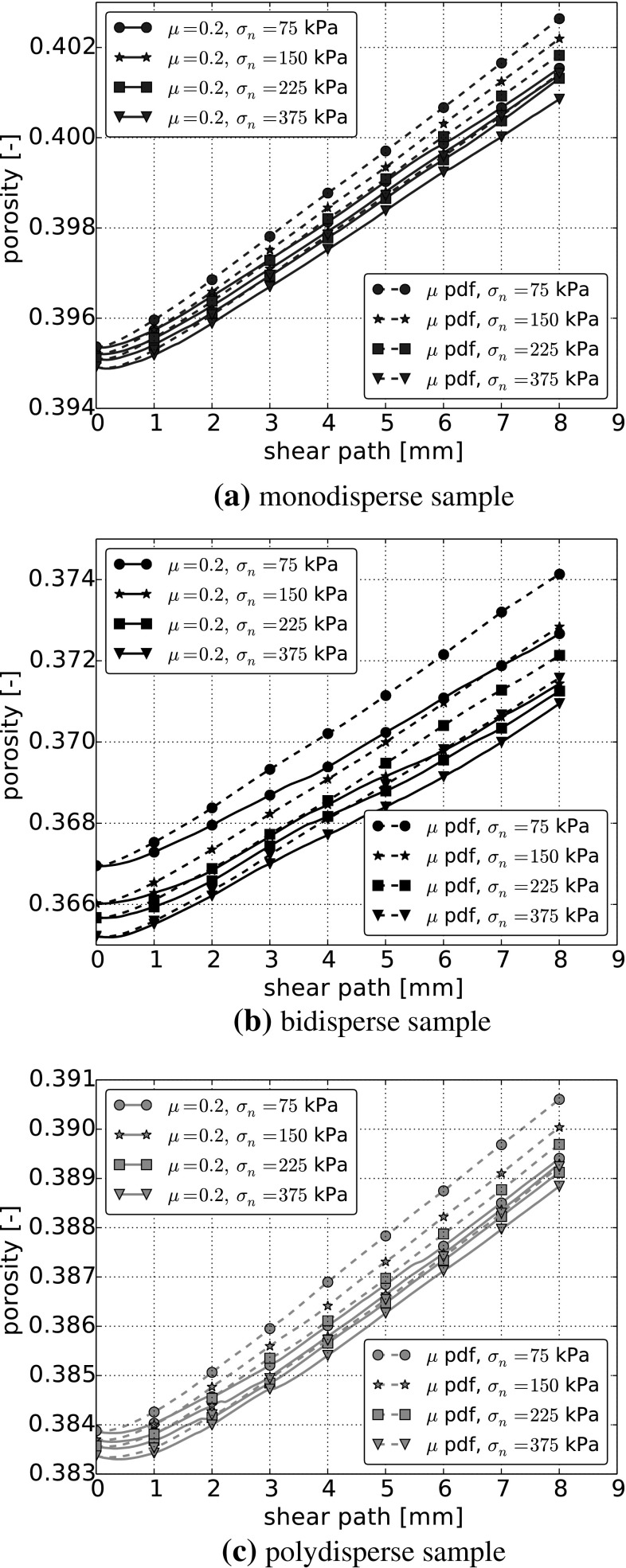
Change in porosity over shear path for simulations with constant and stress-dependent friction coefficient

The authors would like to emphasise that it is not possible to use a higher (constant) friction coefficient and to obtain the same results as with stress-dependent $\mu$. While it would be possible to chose a higher value for interparticle friction such that the final shear stress for $\sigma _\mathrm{n}=75$ kPa is met, the final shear stress for $\sigma _\mathrm{n}=225, 375$ kPa would be too high.

In Fig. 10, histograms of the averaged normal stress, $\bar{\sigma }_\mathrm{n}$, and the stress-dependent $\mu$ from all sphere–sphere contacts are shown. The values are given at the end of the simulation for the lowest level of applied normal stress, $\sigma _\mathrm{n}=75$ kPa. Low averaged normal stresses, $\bar{\sigma }_\mathrm{n}$, belong to high values of interparticle friction and vice versa. While the values of averaged stress and interparticle friction coefficient are in the same range for the three different particle size distributions, the shape of the histograms differ. To calculate the averaged normal stress, $\bar{\sigma }_\mathrm{n}$, the normal force as well as the equivalent contact radius is used. While there is only one equivalent radius in sphere–sphere contacts in the monodisperse sample, there are already three in the bidisperse sample (two small spheres, smalls sphere and big sphere, two big spheres) and in polydisperse samples many different equivalent radii exist. This contributes, together with different distributions of normal forces, to the shapes of the distribution for the interparticle friction coefficient. All three particle size distributions show averaged stresses in the range of 0 GPa to 2.5 GPa. For steel, it can be considered very unlikely that the interparticle friction coefficient will be constant over this wide range of stresses.

The proposed new contact model does affect also the porosities of the samples. In Fig. 11 the porosity is plotted over the shear path for simulations with $\mu =0.2$ and stress-dependent $\mu$. For all three particle size distributions, it can be seen that the samples show considerably more dilation, when the stress-dependent $\mu$ is used in the simulations. With interparticle friction and sample porosity, key factors for the bulk behaviour of a granular material are influenced by the new contact model.

## Conclusions

In this paper, simulations of direct shear tests on monodisperse, bidisperse and polydisperse samples of steel spheres are conducted on four different levels of normal stress, $\sigma _\mathrm{n}= 75, 150, 225, 375$ kPa. For frictional contacts, it is known from tribology that the coefficient of friction is not constant but depends on several factors, such as contact normal load, relative motion, surface roughness, contact temperature and contact conditions (dry, wet, lubricated), etc. Thus, whenever one of these influence factors varies over a wide range, it can be expected that Coulomb’s law, with its one constant coefficient of friction, is an oversimplification of reality. For steel it is known that a dependency of the coefficient of friction on the contact normal load exists. Motivated by results on the wheel–rail contact of steel, a more tribological tangential contact law is implemented in DEM, where the interparticle friction coefficient depends on the averaged normal stress in the contact. The model for the normal load dependency of the interparticle friction coefficient is taken from [12], where model parameters for steel are also provided.

At first, the interparticle friction coefficient is set constant, $\mu =0.2$, and the influence of the samples’ initial configuration is investigated. For the different particle size distributions, deviations from the mean value in final shear stress up to 6 % are seen for five different initial settings. Although the particle size distributions differ considerably for monodisperse, bidisperse and polydisperse samples, the resulting shear stresses are found to be surprisingly similar. The strong influence of interparticle friction on the bulk friction of the granular material is shown via a variation of a constant interparticle friction coefficient. Then, direct shear tests with stress-dependent interparticle friction and constant $\mu =0.2$ are compared. The stress dependency introduced in interparticle friction is clearly seen in the resulting bulk friction coefficient. For the lowest level of applied normal load, $\sigma _\mathrm{n}=75$ kPa, the bulk friction coefficient is larger for stress-dependent $\mu$ than for $\mu =0.2$. For the stress-dependent $\mu$, the bulk friction coefficient decreases with increasing $\sigma _\mathrm{n}$ until it nearly coincides at $\sigma _\mathrm{n}=375$ kPa (where the model was calibrated) with the value obtained with $\mu =0.2$. The extent of this stress dependency is different for monodisperse, bidisperse and polydisperse samples. Also, scatter between results belonging to different initial configuration within each particle size distribution occurs. Nevertheless, the effect of stress-dependent bulk friction is present in all cases. In the literature, e.g. [7, 9], a normal stress dependency of the bulk friction coefficient was seen for some granular materials and small normal loads. Using the proposed stress-dependent interparticle friction coefficient for the simulation of steel spheres, a normal stress dependency of the bulk friction coefficient is also obtained. With increasing normal stress, the bulk friction coefficient reduces both in experiments and in simulations. This effect is not present, when a constant interparticle coefficient is used. Therefore, it is concluded that using the proposed model is one possibility to integrate the normal stress dependency of the bulk friction coefficient in a DEM simulation. It remains future work to conduct experiments for a direct comparison between simulation and measurements.
